# Multi-Fidelity Surrogate-Based Process Mapping with Uncertainty Quantification in Laser Directed Energy Deposition

**DOI:** 10.3390/ma15082902

**Published:** 2022-04-15

**Authors:** Nandana Menon, Sudeepta Mondal, Amrita Basak

**Affiliations:** Department of Mechanical Engineering, The Pennsylvania State University, University Park, State College, PA 16802, USA; nfm5316@psu.edu (N.M.); sudeepta979@gmail.com (S.M.)

**Keywords:** laser-directed energy deposition, melt pool, process maps, multi-fidelity Gaussian process, Bayesian Optimization, uncertainty quantification

## Abstract

A multi-fidelity (MF) surrogate involving Gaussian processes (GPs) is used for designing temporal process maps in laser directed energy deposition (L-DED) additive manufacturing (AM). Process maps are used to establish relationships between the melt pool properties (e.g., melt pool depth) and process parameters (e.g., laser power and scan velocity). The MFGP surrogate involves a high-fidelity (HF) and a low-fidelity (LF) model. The Autodesk Netfabb${}^{®}$ finite element model (FEM) is selected as the HF model, while an analytical model developed by Eagar-Tsai is chosen as the LF one. The results show that the MFGP surrogate is capable of successfully blending the information present in different fidelity models for designing the temporal forward process maps (e.g., given a set of process parameters for which the true depth is not known, what would be the melt pool depth?). To expand the newly-developed formulation for establishing the temporal inverse process maps (e.g., to achieve the desired melt pool depth for which the true process parameters are not known, what would be the optimal prediction of the process parameters as a function of time?), a case study is performed by coupling the MFGP surrogate with Bayesian Optimization (BO) under computational budget constraints. The results demonstrate that MFGP-BO can significantly improve the optimization solution quality compared to the single-fidelity (SF) GP-BO, along with incurring a lower computational budget. As opposed to the existing methods that are limited to developing steady-state forward process maps, the current work successfully demonstrates the realization of temporal forward and inverse process maps in L-DED incorporating uncertainty quantification (UQ).

## 1. Introduction

**Figure 1:**
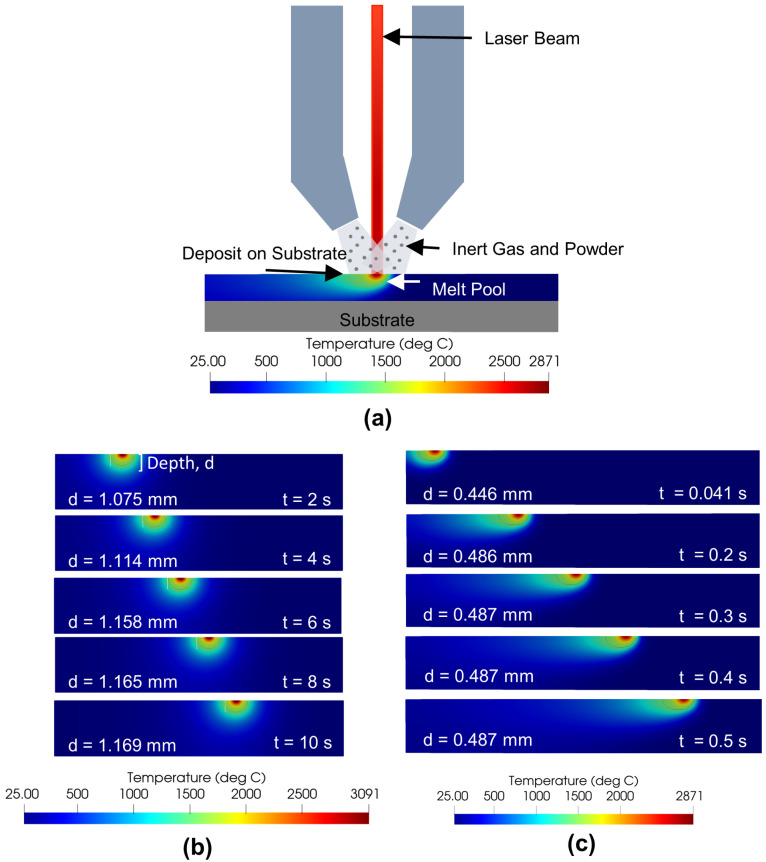
(**a**) A schematic of the L-DED process. (**b**) The melt pool evolution during the L-DED process for a representative power, *P* = 1000 W, and scan velocity, *v* = 1 mm/s showing a near-symmetric melt pool. (**c**) A second representative melt pool evolution at *P* = 1000 W, *v* = 25 mm/s showing the capability of Netfabb^®^ in capturing an asymmetric melt pool evolution. The melt pool reaches steady-state much earlier in (**c**) than (**b**) due to higher *v*.

Laser-directed energy deposition (L-DED) offers tremendous opportunities in manufacturing metallic components because of its ability to fabricate three-dimensional near-net-shape parts with location-specific materials and microstructures [1]. Additionally, L-DED has been used to repair components made of different metallic materials such as steel [2], nickel [3], and titanium [4] alloys. In L-DED, powder or wire feedstocks are delivered at the desired location. The feedstock is melted by a high-power laser heat source as shown in Figure 1a. Process parameters such as the laser power, laser/substrate relative velocity (traverse speed or scan speed), and feedstock feed rate can be varied to achieve the desired deposit quality. The melt pool (as shown in Figure 1b,c), formed by the high-power laser source, plays a critical role in controlling the final microstructure of the L-DED part [5]. For example, investigations have revealed that high energy density produced larger melt pools in L-DED [6].

The melt pool shape and sizes are also affected by environmental conditions and part geometry in L-DED. For example, both simulations and experiments have proven that the presence of surfactants was a critical factor in dictating the shape of the melt pool as the surfactants were found to affect the surface tension [7]. The scan pattern was also found to affect the shape and size of the melt pool due to heat transfer across adjacent tracks and/or layers. Due to these parameters, the melt pool size may vary both in time and space during the L-DED process. Such a variation can detrimentally affect microstructures and, therefore, the mechanical properties of the part. Hence, the impact of toolpath on the melt pool geometry was also investigated [8].

Experimental investigation for evaluating the impact of these different process parameters on the melt pool properties is rather expensive. Moreover, since the thermophysical properties of the metallic materials are drastically different from one another, such an investigation has to be performed for each material of interest. Computational modeling can provide a much-required alternative for estimating the process parameters in L-DED. However, the process parameter development would still require a thorough exploration of the parameter space involving a design-of-experiment (DoE) approach. Computational investigation of such a DoE can be expensive if high-fidelity models are used for predicting the melt pool properties. Hence, it is critical to develop efficient methods for generating process maps that would efficiently estimate the melt pool properties as a function of the process parameters.

Despite recent advances in computational modeling-based process mapping of L-DED additive manufacturing (AM) processes, much of the existing work is focused on developing forward maps, i.e., estimating and predicting the steady-state melt pool characteristics (e.g., melt pool depth and width) as functions of process parameters (e.g., energy density, power, scan speed, and scan pattern, amongst others) or dimensionless numbers [9]. While such studies are extremely useful in understanding the impact of processing parameters on the melt pool properties, they suffer from four critical drawbacks:

- They are computationally expensive when high-fidelity (HF) models are used and less capable when low-fidelity (LF) models are used [10].
- They are deterministic and cannot define the prediction uncertainties when the simulation data is not available for the process parameters of interest [11].
- They are typically used to generate steady-state forward maps although the evolution of melt pool is a transient phenomenon as depicted in Figure 1b,c.
- They are seldom combined with computationally efficient optimization routines toward solving the inverse problem.

To address these limitations, one possibility is to blend the information obtained from the HF and LF simulation models using machine learning tools to develop a multi-fidelity (MF) [12] surrogate. The MF surrogate, thereafter, can be used as a proxy for the prediction of the melt pool properties as a function of process parameters at discrete time instants facilitating the design of temporal process maps. MF surrogates have been demonstrated to efficiently incorporate the information present in a hierarchy of varied fidelity models [13,14] to develop inexpensive estimates of the properties of interest. Such surrogates have been used frequently within the framework of co-kriging [15], where an LF model output acts as an auxiliary data source to enhance the prediction of an HF model [16]. This method is particularly useful when the HF model is computationally very expensive and difficult to evaluate. A modification of this approach, known as recursive co-kriging has been recently developed by Le Gratiet et al. [17,18]. This approach has eventually been used by Perdikaris et al. [19] to tackle the ’curse of dimensionality’ while dealing with large datasets. One of the advantages of using MF surrogates is that they can be utilized as black box optimizers. Such optimizers have been used in a wide variety of engineering design problems [20,21,22,23]. However, such strategies have sparsely been explored in AM, particularly for optimizing the melt pool geometry.

Inspired by the existing research gap, this paper starts by implementing a multi-fidelity Gaussian process (MFGP) surrogate to design the forward process maps for the L-DED AM process at several discrete time instants of the simulation, thereby enabling the design of temporal process maps. Two different melt pool evolution models, namely, the Autodesk Netfabb${}^{®}$ finite element model (FEM) [24] and the Eagar-Tsai [25] analytical model are chosen as the HF and LF ones, respectively. The MFGP surrogate predicts the melt pool depths with uncertainty for process parameters when the true depth is not known. The results demonstrate that the surrogate is capable of predicting the melt pool depth in L-DED at a level of accuracy comparable to a representative HF model, but at a fraction of its computational cost. Once the forward map is designed, the MFGP surrogate is integrated with a Bayesian Optimization (BO) algorithm to design an inverse process map. To demonstrate the efficacy of the inverse map, a case study is performed to obtain the desired melt pool depth under computational budget constraints. The optimal process parameters are found using an approach based on active learning (AL) [26]. Being a Bayesian machine learning framework, the posterior predictions are probabilistic, and are described by an output distribution (as opposed to single-valued estimates obtained from conventional deterministic regression models), which offers a principled methodology of uncertainty quantification (UQ) [27]. The performance of multi-fidelity Bayesian Optimization (MFGP-BO) is compared with single-fidelity Bayesian Optimization (SFGP-BO). SFGP-BO utilizes the HF model as the sole information source. The results demonstrate that MFGP-BO can not only find the optimal process parameters faster than SFGP-BO but also with an improved quality (defined as the percent deviation from the desired melt pool depth).

The paper is arranged in four sections, including the present one. The methodology section outlines the thermal models for melt pool prediction, MF surrogate development, and Bayesian Optimization. The next section presents the results, followed by a conclusion section outlining the applications and future perspective of the research. All simulations reported in this paper are conducted on an Intel^®^ Xeon^®^ Gold 6230 CPU processor with 128 GB of RAM.

## 2. Methods

### 2.1. Thermal Models for Melt Pool Predictions

Several different simulation models are available in the open literature for the prediction of melt pool properties in L-DED. Eagar and Tsai [25] improved Rosenthal’s [28] analytical model by replacing the point heat-source with a Gaussian distribution. The Eagar-Tsai model has been widely employed to conduct rapid simulations for a wide range of materials. An analytical model involving metal fluid flow and mass transfer in addition to heat transfer in L-DED was developed by Gan et al. [29]. The solidus and liquidus thermal properties were considered, thereby, capturing the Marangoni convection which showed a strong influence on the melt pool shape [30]. This model was experimentally validated for single-layer IN718 deposits on AISI 1045 carbon steel [31]. Huang et al. [32] developed a comprehensive analytical model, from Rosenthal’s 3D temperature distribution, by coupling both mass and energy flows. This model accommodated for the attenuation of laser power due to the change in clad geometry and powder-gas stream interactions. The model was experimentally validated for single-layer depositions of IN625. The effect of Marangoni flow was incorporated by using a modified thermal conductivity parameter.

While analytical models for L-DED provide valuable melt pool-related information, they include several simplifications. Computational capabilities using finite element models (FEMs) can address these simplifications. Anderson et al. [33] simulated L-DED of nickel-base superalloy CMSX-4^®^ using a model developed by DebRoy [34]. However, it did not consider the variation of material properties with temperature. Kamara et al. [35] used a commercial FEM suite, Ansys Fluent to simulate the transient evolution of melt pool for IN718 powder-fed L-DED. Other commercial software are also available. Autodesk’s Netfabb${}^{®}$ [24] is a software application tailored for AM that has reduced the computation time significantly through adaptive meshing-based FEM techniques [36]. Netfabb${}^{®}$ uses a nonlinear Newton–Raphson-based code and solves for the transient behavior of the L-DED process.

MF surrogates are developed, in this paper, to supplement the available HF predictions with inexpensive LF predictions. Combining data from multiple sources naively may result in biased predictions which do not accurately reflect the physics. Hence, the construction of a reliable MF surrogate depends on the careful selection of its constituent HF and LF models. Based on the existing literature [9], an analytical model (e.g., Eagar-Tsai), due to its many simplifications, shows lower computational cost. On the contrary, an FEM model (e.g., Netfabb^®^), shows higher computational expense but incorporates several modeling parameters (e.g., temperature-dependent thermophysical properties). These two models can define the hierarchical fidelity levels since they are defined by the same governing equations that explain the underlying physical phenomenon, provided they are numerically comparable in their discretized domains (i.e, both models have similar grid sizes). This observation forms the basis for selecting the Eagar-Tsai and Netfabb^®^ models as LF and HF models, respectively, in this paper.

#### 2.1.1. Eagar-Tsai’s Model

**Figure 2:**
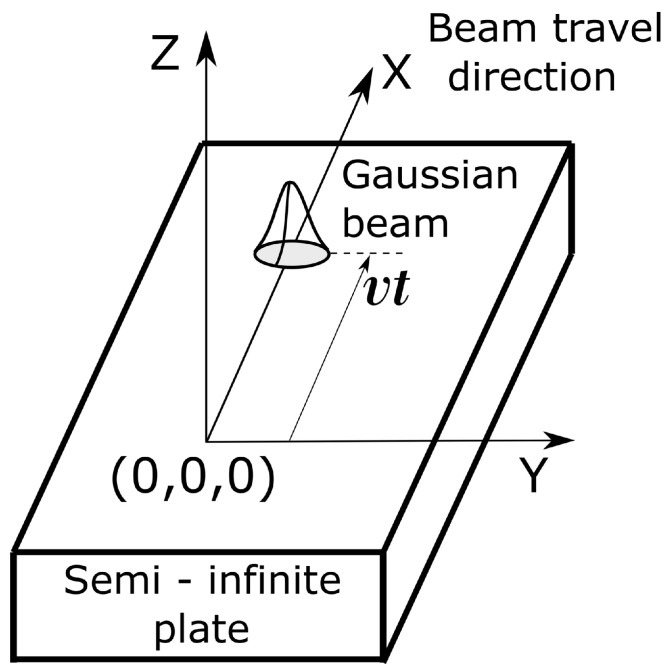
Schematic illustrating the coordinate system of the analytical model. The figure is reproduced from [37] under the terms of the Creative Commons Attribution 4.0 License from http://creativecommons.org/licenses/by/4.0/ (accessed on 9 April 2022).

The LF model used in this paper is the analytical model developed by Eagar and Tsai [25] which solves for the three-dimensional temperature distribution produced by a traveling distributed heat source moving on a semi-infinite plate. This model is a modification of the Rosenthal’s model where a Gaussian heat distribution is used instead of a point source. Figure 2 explains the coordinate system used in the Eagar-Tsai model. The heat source is traveling with a uniform speed of *v* in the *x*-direction, and is assumed to be a 2D surface Gaussian. The temperature $T(x_{c},y_{c},z_{c},t)$, at a particular location $\left(x_{c},y_{c},z_{c}\right)$ and time *t* is calculated as:

(1) $$\begin{array}{c} T\left(x_{c},y_{c},z_{c},t\right)-T_{0}=\frac{\alpha_{L}P}{\pi \rho_{p}c_{p}{\left(4\pi a_{p}\right)}^{1/2}}\int_{0}^{t}\frac{dt^{\prime }{\left(t-t^{\prime }\right)}^{-1/2}}{2a_{p}\left(t-t^{\prime }\right)+{\sigma_{L}}^{2}}e^{-\frac{{\left(x_{c}-vt^{\prime }\right)}^{2}+y_{c}^{2}}{4a_{p}\left(t-t^{\prime }\right)+2{\sigma_{L}}^{2}}-\frac{z_{c}^{2}}{4a_{p}\left(t-t^{\prime }\right)}} \end{array}$$

Here, $T_{0}$ is the initial temperature of the substrate, $\alpha_{L}$ is the absorptivity of the laser beam, *P* is the power, $\rho_{p}$, $k_{p}$, and $c_{p}$ are the density, thermal conductivity, and specific heat capacity of the material, respectively, $a_{p}≜\frac{k_{p}}{\rho_{p}c_{p}}$ is the thermal diffusivity of the material, $t^{\prime }$ is the dummy integration variable, $\sigma_{L}$ is the distribution parameters, and *v* is the scan velocity. Similar to Rosenthal’s equation, the Eagar-Tsai model also makes several assumptions: (i) absence of heat transfer due to convection and radiation; (ii) constant thermal properties for the material; (iii) quasi-steady state semi-infinite medium, and absence of any phase change.

A reasonable agreement between the theoretical and experimental data for steel, titanium and aluminum is demonstrated by Eager and Tsai [25]. This model has been widely used by researchers and has been incorporated with experimental studies for evaluating the melt pool evolution in L-DED processes to develop process mapping strategies.

#### 2.1.2. Autodesk Netfabb^®^ Model

Netfabb^®^ model by Autodesk, a non-linear decoupled 3D transient FEM solver, is used as the HF model [36,38]. The underlying methodology of Netfabb^®^ rests on the decoupled or weakly coupled modeling assumption that the relationship between the thermal and mechanical behaviors are unidirectional so that the thermal history affects the mechanical behavior, but the vice-versa does not hold. The Netfabb^®^ model includes Marangoni convection, convection and radiation heat losses, and the temperature-dependent thermophysical properties omitted by the Eagar-Tsai model.

The model domain is determined based on the scan velocity range so that the domain is large enough in the scan direction such that the melt pool reaches a steady-state. For thermal investigations, the energy balance is the governing equation, which is converted to a weak formulation using the Galerkin approach [39]. The distribution of heat through the part is described by the Fourier’s conduction equation. The model uses a 3D volumetric heat source. The thermal boundary losses include thermal radiation, free convection, and forced convection. The total heat loss flux from the model is thus given by,

(2) $$q=h_{\mathrm{eff}}\left(T_{s}-T_{\infty }\right)$$

(3) $$h_{\mathrm{eff}}=h_{\mathrm{free}}+h_{\mathrm{forced}}+h_{\mathrm{radiation}}$$

Here, $T_{s}$ is the surface temperature, $T_{\infty }$ is the ambient temperature, $h_{\mathrm{eff}}$ is the effective heat transfer coefficient which is a summation of the free convection ($h_{\mathrm{free}}$), forced convection ($h_{\mathrm{forced}}$), and radiation ($h_{\mathrm{radiation}}$). Free convection arises due to the thermal gradients developed during the L-DED process while forced convection arises from the shielding gas and powder flowing over the melt pool. This model has been experimentally validated for L-DED processes for a popular nickel-base superalloy, IN625 [39] showing excellent agreement between the simulation and experimental data.

### 2.2. Surrogate Development

This subsection briefly presents the mathematical fundamentals of the GP and MF surrogate that integrates the LF and HF thermal models [40,41].

#### 2.2.1. Gaussian Process (GP) Surrogate

The surrogate development strategy is based on a class of stochastic processes called ’GPs’ that assume any finite collection of random variables to follow a multivariate jointly Gaussian distribution. For a finite collection of inputs, x, the corresponding function outputs, y are assumed to have a multivariate jointly Gaussian distribution,

(4) $$y∼N(\mu \left(x\right)\;,k\left(x,x^{\prime }\right))$$

Here, N implies a Gaussian distribution. The underlying GP is completely characterized by a mean function: μ(x)≜E[y], and a covariance function: $k\left(x,x^{\prime }\right)≜E\left[y-\mu \left(x\right)\right)\left(y^{\prime }-\mu \left(x^{\prime }\right)\right)]$ [27]. Here, E[y] denotes the expectation of y. $x^{\prime }$ and $y^{\prime }$ denote an input other than x and the corresponding functional output of it, respectively. In the context of the melt pool prediction, each input point comprises of different (P,v) combinations. The melt pool depths (*d*) corresponding to the (P,v) combinations are jointly Gaussian. The training data-set comprises of $x^{trn}≜\left(P^{trn},v^{trn}\right)$ as the inputs and the known melt pool depths, $y^{trn}≜d^{trn}$ as the outputs. The test data-set comprises of $x^{tst}≜\left(P^{tst},v^{tst}\right)$ as the inputs for which the melt pool depths are unknown. The conditional distribution of the outputs at the test locations is given by:

(5) $$y^{tst}|y^{trn},x^{trn},x^{tst}∼N\left(\mu^{tst},\Sigma^{tst}\right)$$

Here,

(6) $$\begin{array}{c} \mu^{tst}=K\left(x^{tst},x^{trn}\right){\left[K\left(x^{trn},x^{trn}\right)+{\sigma_{\epsilon }}^{2}I\right]}^{-1}y^{trn} \end{array}$$

(7) $$\begin{array}{c} \Sigma^{tst}=K\left(x^{tst},x^{tst}\right)-K\left(x^{tst},x^{trn}\right){\left[K\left(x^{trn},x^{trn}\right)+{\sigma_{\epsilon }}^{2}I\right]}^{-1}K\left(x^{trn},x^{tst}\right) \end{array}$$

Here, I is the identity matrix and K is the covariance matrix. Thus, the predicted posterior distribution of the outputs at every test data point is also a Gaussian distribution, characterized by the mean, $\mu^{tst}$ and covariance, $\Sigma^{tst}$. A detailed mathematical account of GPs can be found in  [27].

#### 2.2.2. Multi-Fidelity (MF) GP Surrogate

Often computational models present a hierarchy of fidelities for a given process. HF models are generally more capable, but expensive. On the other hand, LF models are typically less capable, but cheaper. To develop process maps, an extensive use of HF models can be computationally infeasible. In such cases, it would be judicious to adopt intelligent strategies that leverage the computational inexpensiveness of the LF models by using them more frequently.

**Figure 3:**
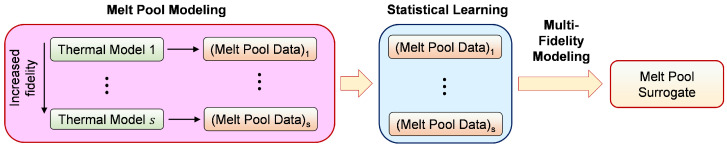
Presence of a hierarchy of fidelities in modeling platforms. Statistical learning integrates multi-fidelity (MF) information.

MF surrogate is a statistically-learned framework [12] that integrates the information present in all fidelities to develop a ’proxy’ which can predict outputs with the accuracy of HF models, but with significantly inexpensive computational overhead. The general structure of MF information source is shown in Figure 3, which shows several levels of fidelities in the models. Such a framework relies on data-driven learning of the correlation among the different fidelities. Co-kriging approaches have been studied extensively for performing the joint estimation of the outputs from correlated variables [12,13,14]. The co-kriging approach employed in this work is based on the autoregressive formulation of Keneddy and O’Hagan [15]. MFGPs using co-kriging approaches rely on formulating separate surrogates for each fidelity which are coupled together through an appropriate covariance function in a GP setting.

For an input set of process parameters, x, the autoregressive formulation for 2 fidelities is given by:

(8) $$y_{2}=\rho y_{1}+\delta \left(x\right)$$

Here, $y_{1}$ is the low-fidelity output and $y_{2}$ is the high-fidelity output. ρ quantifies the correlation between $y_{2}$ and $y_{1}$. The Gaussian process, δ(x) represents the discrepancy between $y_{1}$ and $y_{2}$. These outputs, $y_{1}$ and $y_{2}$, take the jointly Gaussian distribution of the form:

(9) $$\begin{array}{c} Y=\left[\begin{array}{c} y_{1}\\ y_{2} \end{array}\right]∼N\left(0,\left[\begin{array}{c} k_{1}\left(x_{1},x_{1}^{\prime };\theta_{1}\right)+\sigma_{\epsilon_{1}}^{2}I\;\;\rho k_{1}\left(x_{1},x_{2}^{\prime };\theta_{1}\right)\\ \rho k_{1}\left(x_{2},x_{1}^{\prime };\theta_{1}\right)\;\;\;\rho^{2}k_{1}\left(x_{2},x_{2}^{\prime };\theta_{1}\right)+\\ \;\;\;\;\;\;\;k_{2}\left(x_{2},x_{2}^{\prime };\theta_{2}\right)+\sigma_{\epsilon_{2}}^{2}I \end{array}\right]\right) \end{array}\;$$

Here, $k_{1}$ and $k_{2}$ are the kernel functions, $\sigma_{\epsilon_{1}}^{2}$ and $\sigma_{\epsilon_{2}}^{2}$ are the noise levels, $\theta_{1}$ and $\theta_{2}$ are the hyperparameters, where the subscripts 1 and 2 correspond to LF and HF models, respectively. The Negative Log Marginal Likelihood (NLML) in a two-fidelity setting is given by:

(10) $$-\log\;p\left(Y|X,\theta_{1},\theta_{2},\rho ,\sigma_{\epsilon_{1}}^{2},\sigma_{\epsilon_{2}}^{2}\right)=\frac{1}{2}\log\left|K\right|+\frac{1}{2}Y^{T}K^{-1}Y-\frac{N_{L}+N_{H}}{2}\log2\pi$$

Here, **X** and **Y** are the combined observed inputs and their outputs from the LF and HF models, respectively. $N_{L}$ and $N_{H}$ correspond to the number of observed input-output data from LF and HF models, respectively. The MFGP methodology is outlined in Algorithm 1.
**Algorithm 1** Multi Fidelity Gaussian Process**Require:** Low-fidelity input, $x_{1}$; Low-fidelity output, $y_{1}$; Hyperparameter of the low fidelity kernel, $\theta_{1}$; High-fidelity input, $x_{2}$; High-fidelity output, $y_{2}$; Hyperparameter of the high fidelity kernel, $\theta_{2}$; Kernel function, *k* (for simplicity, here $k_{1}$ = $k_{2}$); Test input, $x^{tst}$; Noise-level, $\sigma_{\epsilon }^{2}$ (for simplicity, here $\sigma_{\epsilon_{1}}^{2}$ = $\sigma_{\epsilon_{2}}^{2}$)
1:**L** = cholesky(**K** +$\sigma_{\epsilon }^{2}$**I**) ▹ **K** as calculated from Equation (9)2:**Y** = [$y_{1}\;y_{2}$]3:α = $L^{T}\setminus \left(L\setminus Y\right)$4:$\psi_{1}=\rho k\left(x^{tst},x_{1},\theta_{1}\right)$5:$\psi_{2}=\rho^{2}k\left(x^{tst},x_{2};\theta_{1}\right)+k\left(x^{tst},x_{2};\theta_{2}\right)$6:$\Psi =[\psi_{1}\;\psi_{2}]$7:${\hat{f}}_{x^{tst}}=\Psi .\alpha$ ▹ predictive mean8:$\beta =L^{T}\setminus \left(L\setminus \Psi^{T}\right)$9:$V\left[f_{x^{tst}}\right]=\rho^{2}k\left(x^{tst},x^{tst},\theta_{1}\right)+k\left(x^{tst},x^{tst};\theta_{2}\right)-\Psi \beta$ ▹ predictive variance


The kernel function at every fidelity level has its own hyperparameter. The choice of the kernel function is a critical element for the success of a GP algorithm since it encodes the correlation between the points in the feature space. Typically, squared exponential kernels are best suited for interpolating smooth functional relationships. However, in this paper, Matérn kernels, with shape parameters of 5/2, are used since their length-scales are less prone to be affected by non-smooth regions, thereby improving performance in these regions. [40,42]. The MFGP is learnt by optimizing the hyperparameters through minimizing NLML using the Limited-memory Broyden–Fletcher–Goldfarb–Shanno (L-BFGS) algorithm [43]. While the current formulation focuses on a two-fidelity setup, this can be extended to higher fidelities by making appropriate modifications in the covariance matrix which correlates different levels of fidelity [15].

### 2.3. Bayesian Optimization (BO)

This subsection briefly presents the mathematical fundamentals of the Bayesian Optimization for single-fidelity (SF) and multi-fidelity (MF) surrogates [40,41].

#### 2.3.1. Single-Fidelity Gaussian Process with Bayesian Optimization (SFGP-BO)

In this description, the term *optimization* is used to denote maximization of an objective function. A minimization problem can be posed similarly by taking the negative of the objective function. In a single-fidelity setting, there is a single objective function *f*. To optimize *f* over its domain, the solver needs to find:

(11) $$\hat{x}=\underset{x\in X^{*}}{\mathrm{argmax}}f\left(x\right)$$

Here, ’argmax’ finds the argument that gives the maximum value from an objective function, *f*. The functional form of *f* is typically unknown and, hence, a gradient-free or *black-box* optimization is often utilized. BO is one such *black-box* optimization technique [44] that leverages the predictions through a surrogate for sequential *active learning* to find the global optima of the objective function. The AL strategies find a trade-off between *exploration* and *exploitation* in possibly noisy settings [45,46,47], which facilitates a balance between the global search and local optimization through *acquisition functions*. One commonly used acquisition function in BO is Expected Improvement (*EI*). A detailed formulation of EI can be found in the work of Mockus et al. [48] and Jones et al. [45].

The objective function, f(x) is often represented as a GP, which yields a posterior predictive Gaussian distribution characterized by the mean μ(x) and standard deviation σ(x) for $x\in X^{*}$, where $X^{*}$ is the search space of the optimization challenge. When the optimization framework involves a single-fidelity GP, it is referred to as single fidelity GP (SFGP)-based optimization. The optimization algorithm proceeds sequentially by sampling $\hat{x}={argmax}_{x}EI\left(x\right)$ at every step of the iteration process to add on to the dataset, after which the GP surrogate is retrained with the new data set to predict the acquisition potential for the next iterative step. This process continues until an optimum is reached, or the computational budget is extinguished. Since the acquisition potential is predicted over the entire search space by the surrogate, BO can achieve fast predictions without a lot of function calls in the search space (i.e., without having to run the simulations to obtain the objectives at all the search locations). This process otherwise, might be computationally infeasible when the search space is high-dimensional and the simulations are expensive.

#### 2.3.2. Multi-Fidelity Gaussian Process with Bayesian Optimization (MFGP-BO)

In the presence of multiple levels of fidelities, the recent work of Sarkar et al. [40] presents a demonstration of choosing appropriate acquisition functions for the HF and LF models to guide the search for optimum using MF surrogates. The HF predictions are chosen using the constrained EI acquisition function, while the LF predictions are selected using the GP-based Mutual Information Acquisition function (GP-MI) [49]. The rationale behind employing GP-MI for LF selections is in its mathematical formulation: GP-MI would preferentially promote *exploration* at the initial stages of optimization, and gradually drive *exploitation* as the global prediction becomes progressively more accurate in the subsequent iterations. Since a majority of engineering applications involves expensive and possibly limited HF evaluations, GP-MI fits the requirements of being exploratory at the initial stages where inexpensive LF evaluations can be leveraged to learn the process parameter space [40]. The algorithm implemented for MFGP-BO methodology is outlined in Algorithm 2.

**Algorithm 2** MFGP—Bayesian Optimization**Require:** Search space for optimization, $X^{*}$; MFGP prior with mean function, μ(x) and kernel function, $k(x,x^{\prime })$; objective function, J(t,P,v); number of optimization steps allowed, $N_{\mathrm{opt}}$; $N_{H},N_{L}$, accepted tolerance for the melt pool depth, ϵ
1:**for**i←1 to $N_{\mathrm{opt}}$ **do**2:    **if** $|d\left(t,P,v\right)-d^{*}|<\epsilon$ **then**3:        Perform HF simulations at $\hat{x}={argmax}_{x}EI\left(x\right)$4:        Augment data with HF predictions, update MFGP5:        Perform LF simulations at $\hat{x}={argmax}_{x}GP-MI\left(x\right)$6:        Augment data with LF predictions, update MFGP7:    **else**8:        **break**9:    **end if**10:**end for**


## 3. Results and Discussion

### 3.1. Melt Pool Predictions from Thermal Models

Single crystal nickel-based superalloys have been increasingly deployed in gas turbines owing to their excellent high-temperature properties. CMSX-4^®^ is one such popular second-generation ultra high-strength superalloy that is selected as the candidate alloy in this paper [50]. The life-cycle of these expensive parts comprising CMSX-4^®^ is often limited by blade tip wear and crack, thereby requiring a feasible method of repair that ensures directional solidification in the repaired zones. L-DED has proved to be successful in achieving this objective [51]. A major determinant of the growth of SX epitaxial layers during L-DED is the laser process parameters. A lower laser power can increase the thermal gradient and promote epitaxial growth. A higher laser scanning velocity yields a shallow melt pool and stimulates epitaxial growth [52]. This outlines a critical need to propose processing windows for the manufacture and/or repair by L-DED. This section summarizes the results obtained from the low- and high-fidelity thermal models used to simulated the single scan deposits of CMSX-4^®^.

#### 3.1.1. Predictions from LF Eagar-Tsai Model

The thermo-physical properties of CMSX-4${}^{®}$ chosen for the LF model are those reported by Gäumann et al. [51]: $k_{p}$ = 22 W/(m·K), $\rho_{p}$ = 8700 kg/m^3^, $c_{p}$ = 690 J/(kg·K), and the liquidus temperature $T_{L}$ = 1660 K. Other modeling parameters and their selected values are: $T_{\infty }$ = 25 ${}^{\circ }$C and $\alpha_{L}$ = 0.35. The laser beam radius is maintained at 0.39 mm.

**Figure 4:**
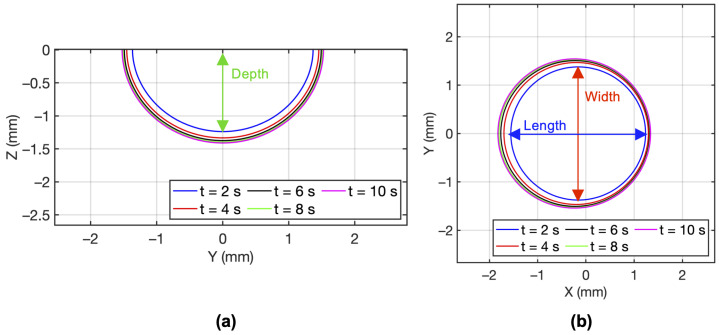
Temporal variation of the (**a**) melt pool depth and (**b**) melt pool length and width, for *P* = 1000 W and *v* = 1 mm/s.

Figure 4 shows the two-dimensional melt pool at different time instants illustrating the temporal nature of the melt pool evolution for laser power, *P* = 1000 W and scan velocity, *v* = 1 mm/s. The results are also reported in Table 1. The melt pool reaches a steady-state after *t* = 20 s. Similar behavior is observed for other *P* and *v* combinations as well.

#### 3.1.2. Predictions from HF Netfabb^®^ Model

**Figure 5:**
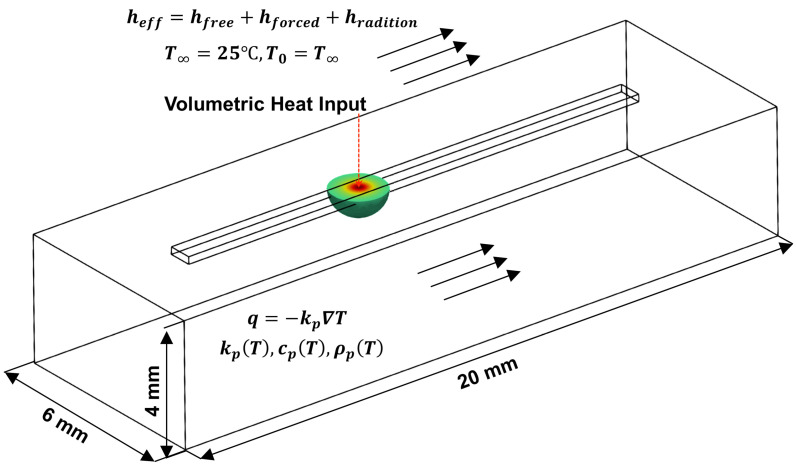
Simulation domain with boundary conditions.

Single-track single-layer simulations are performed using the Netfabb^®^ model. The simulation domain is shown in Figure 5. The laser parameters, i.e., laser radius and absorptivity, and the ambient temperature are kept identical to the Eagar-Tsai model. The effective heat transfer coefficient is set as $h_{\mathrm{eff}}$ = 25 W/m${}^{2}$C [53]. The temperature of the substrate plate is kept at 25 ${}^{\circ }$C. The laser vector file containing the laser power, laser vector, start and end positions of the laser, laser radius, scan speed, and start time, is fed into the Netfabb^®^ model before creating the simulation domain. The temperature-dependent properties for CMSX-4^®^ are obtained from JMatPro^®^ [54] and shown in Table 2.

**Figure 6:**
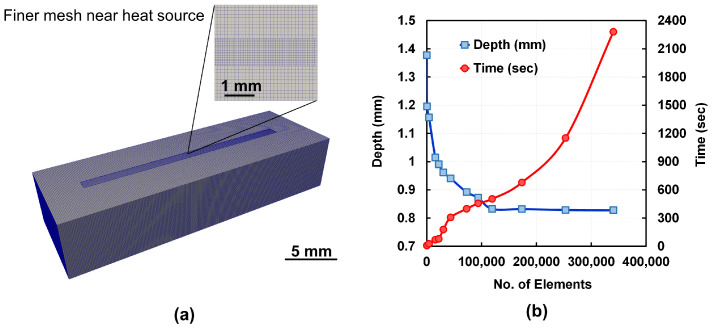
(**a**) Adaptive meshing applied to the simulation domain. (**b**) The variation of the melt pool depth and the simulation time with the number of mesh elements.

The L-DED-specific mesh features are varied to implement adaptive meshing which reduces the number of mesh elements [55] away from the melt pool as shown in Figure 6a. These features are (i) the number of elements per heat source radius, (ii) the number of fine layers below the heat source, and (iii) the number of mesh adaptivity levels. A mesh convergence test is conducted by varying the quality of the mesh for the simulation domain described in Figure 5. The difference between the melt pool depths calculated from the model having the finest mesh (340,400 elements) and the selected model (119,392 elements) is 0.54%, which is lesser than the minimum of 5% specified by Netfabb^®^ for a good mesh convergence [53]. Figure 6b shows the results obtained from the mesh convergence study.

**Figure 7:**
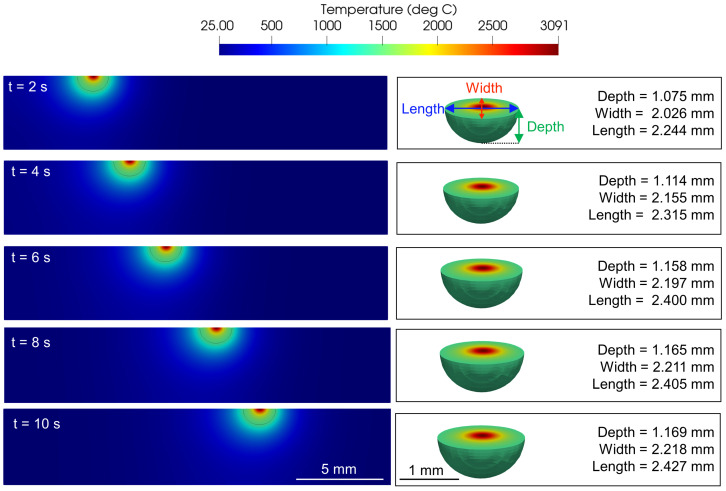
ZX cross section of the model simulations and the corresponding extracted isovolume to its right, showing the temporal variation of melt pool dimensions *P* of 1000 W and scan velocity *v* of 1 mm/s.

The melt pool properties are obtained from the Netfabb${}^{®}$ model using ParaView [56], an open-source, multi-platform data analysis and visualization application. The thermal models are post processed to extract an isovolume corresponding to the melt pool that is defined by the liquidus temperature of the material i.e., $T_{L}$= 1660 K for CMSX-4${}^{®}$. Figure 7 shows the evolution of melt pool at four different time instants for *P* = 1000 W and *v* = 1 mm/s with the corresponding three-dimensional melt pool volume extracted. The results obtained from the Netfabb${}^{®}$ model also reinstate the temporal nature of the melt pool necessitating the requirement of temporal process maps.

#### 3.1.3. Comparison of Melt Pool Properties—Eagar-Tsai vs. Netfabb^®^

**Figure 8:**
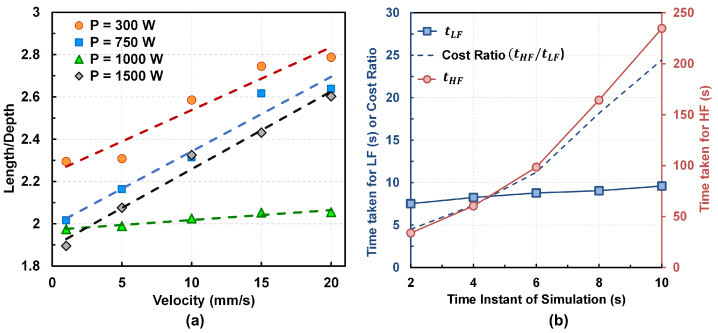
(**a**) The length to depth ratio of the melt pool for different combinations of laser power and velocity. Similar relationships are also observed for the melt pool length and depth and, hence, not shown here for brevity. (**b**) Variation of the cost ratio of HF to LF model at several discrete time instants of the melt pool simulation. Here $t_{LF}$ is the time taken by LF simulations and $t_{HF}$ is the time taken by HF simulations.

While in practice, the ranges of *P* and *v* depend on the experimental apparatus, a higher scan velocity will require a larger domain in the scan direction to ensure that the melt pool has reached a steady-state, which, consequently, will increase the cost of simulation of the Netfabb^®^ model. The relationships between the the melt pool geometry (e.g., length, depth, and length to depth ratio) and the scan velocity over a wide range of power is shown in Figure 8a. The steady state melt pool length and depth are calculated using Netfabb^®^ for CMSX-4^®^. This near-linear behavior indicates that the conclusions obtained from the current study will be applicable to other domain sizes as well. It is found that the simulation domain size of 22 mm × 6 mm × 4 mm accommodates the *P* range of (300 W–1300 W) and *v* range of (0.1 mm/s–1.5 mm/s) selected for the process parameter space, $X_{space}$.

The LF Eager-Tsai model rapidly conducts simulations over the process parameter space compared to the HF Netfabb^®^ model. Figure 8b shows the time taken by each LF and HF model, and the ratio of time taken by the HF model to the LF model for each discrete time instants of the melt pool simulation. In this study, the cost of the Eagar-Tsai model is the time taken to solve the temperature over a three-dimensional domain that has been divided into 100 equal divisions in each direction. The cost of the Netfabb^®^ model pertains to the total simulation time for the final mesh selected after the mesh convergence study.

### 3.2. Design of Forward Process Maps

**Figure 9:**
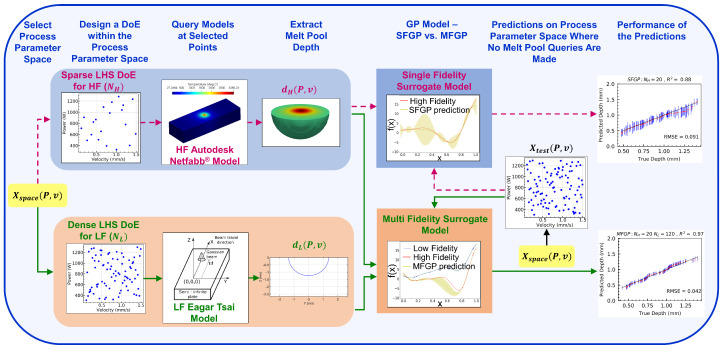
Framework followed for developing the forward process maps at discrete time instants using SFGP/MFGP. The solid green line indicates the path of the MFGP regression while the dashed magenta line indicates the path of the SFGP regression.

The overall methodology for developing the forward process maps is shown in Figure 9. The first step toward developing a process map is to perform an Latin Hypercube Sampling (LHS) [57] in the input data space, $X_{space}$. Each input data point is a combination of (P,v) values. Each output data point is the corresponding melt pool depths, $d_{H}$ (depth obtained from HF model) and $d_{L}$ (depth obtained from LF model).

The MFGP surrogate is, thereafter, formulated as follows:

- LHS is employed to select $N_{L}$ input data points in $X_{space}$ for which the LF model is used to obtain the steady-state melt pool depths.
- Similarly, $N_{H}$ input data points are selected in $X_{space}$ through a separate LHS, for which the melt pool depths are obtained from the HF model.
- Using $N_{L}$ LF input-output data points and $N_{H}$ HF input-output data points, the MFGP surrogate is trained using the maximum likelihood estimation [12] at each discrete time instants of simulation.

LHS is adopted in this work because it is one of the most commonly used statistical methods for DoE [57], which, by virtue of its high sampling efficiency, is capable of generating a good spread of the initial input data points over the process parameter space within a limited number of iterations. The prediction performance of the trained MFGP surrogate is carried out on a set of *N* test input points ($X_{test}$) in the process parameter space (i.e., $X_{test}$∈$X_{space}$). The predicted values are, then, compared with the true melt pool depths ($d_{true}$) which refer to the outputs from the HF Autodesk Netfabb${}^{®}$ model at $X_{test}$. When the number of LF simulations is 0, the MFGP surrogate essentially becomes an SFGP.

#### 3.2.1. Performance Metrics

To investigate the performance of GP surrogates, the following evaluation metrics are used:

- Root Mean Square Error, RMSE $≜\sqrt{\frac{1}{N}\sum_{i=1}^{N}{\left|d_{pred_{\mu }}\left(i\right)-d_{true}\left(i\right)\right|}^{2}}$. Here, $d_{pred_{\mu }}\left(i\right)$ denotes the mean of the posterior predictive Gaussian distribution at a test input indexed *i* in $X_{test}$. $d_{true}\left(i\right)$ denotes the true melt pool depth at the same test input and *N* is the number of test inputs. Additionally, $RMSE_{avg}$≜RMSE/50, when 50 different DoE initializations are used.
- Total σ of prediction = $\sum_{i=1}^{N}\sigma_{pred}\left(i\right)/50$. Here, $\sigma_{pred}\left(i\right)$ is the standard deviation of the posterior predictive Gaussian distribution at a test input indexed with *i* in $X_{test}$.

#### 3.2.2. Effect of Adding LF Predictions

Each initialization of the MFGP and SFGP surrogates involves a unique choice of the HF and LF input-output data points for the surrogate formulation. This is achieved through the generation of unique input data points for each fidelity model using LHS.

**Figure 10:**
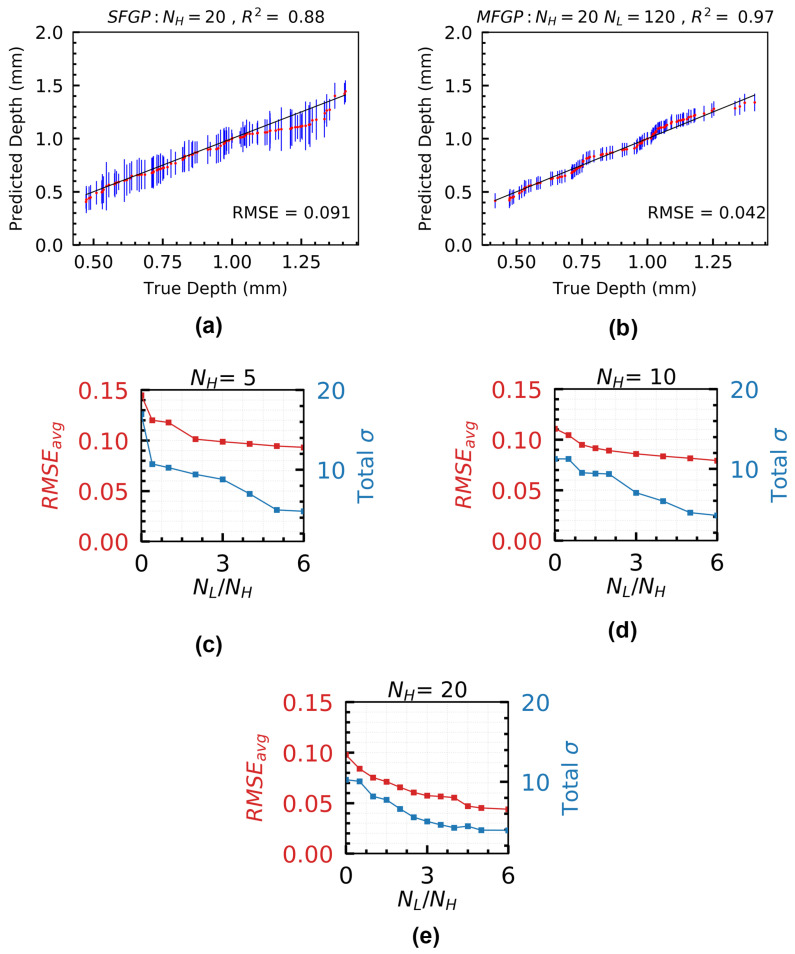
Parity plots for (**a**) SFGP with $N_{H}=20$ and (**b**) MFGP with $N_{H}=20,N_{L}=120$. Variation of $RMSE_{avg}$ and Total σ as a function of $N_{L}$ by keeping $N_{H}$ fixed at (**c**) 5 (**d**) 10, and (**e**) 20. $N_{L}/N_{H}$ has been varied from 0 to 6. The performance of MFGP in predicting the melt pool depth is evaluated on a test set, $X_{test}$ of 100 randomly sampled points from $X_{space}$. The results reported are the corresponding averages of RMSE and σ over 50 different initializations of the MFGP.

Since HF models are typically computationally expensive, it is of critical interest to investigate if adding more LF simulations to an MFGP surrogate with a limited number of HF simulations can improve the prediction performance. Figure 10 shows the effect of adding LF simulations to an MFGP surrogate for which the number of HF simulations ($N_{H}$) is constant. Since, in practice, the predictions are often dependent on the training data choice [58], the regression performance of the MFGP and SFGP surrogates reported in Figure 10 are based on the average performance over multiple initializations of the initial DoE of the respective surrogates. An intentional choice of low $N_{H}$ values is adopted since it is challenging to fit a reasonably good MFGP surrogate when $N_{H}$ is low [40].

Adding data from the LF model results in the reduction of both $RMSE_{avg}$ and total σ of the prediction. A reduction in $RMSE_{avg}$ corresponds to more accurate predictions of the melt pool depth, while a reduction in σ indicates higher confidence in the MFGP surrogate predictions [40]. For $N_{H}=20$, Figure 10c shows a 55% reduction in $RMSE_{avg}$, and 61% reduction in total σ for $N_{L}/N_{H}=6$, as compared to the SFGP surrogate ($N_{L}/N_{H}=0$).

A similar trend is also observed for the other two cases of fixed $N_{H}$ data. The highest reduction in $RMSE_{avg}$ and σ occurs for $N_{H}=20$ as $N_{L}$ is increased. This is due to the presence of a larger amount of HF data in the corresponding MFGP surrogate which is expected to reduce $RMSE_{avg}$. But, even for the case with $N_{H}=5$, the reductions in $RMSE_{avg}$ and total σ are found to be 35% and 71%, respectively. This demonstrates the capability of the MFGP surrogate to efficiently incorporate MF information to improve the predictive performance as well as to achieve higher confidence in the predictions in a Bayesian setting, without increasing the total simulation cost significantly.

#### 3.2.3. Effect of Adding HF Predictions

**Figure 11:**
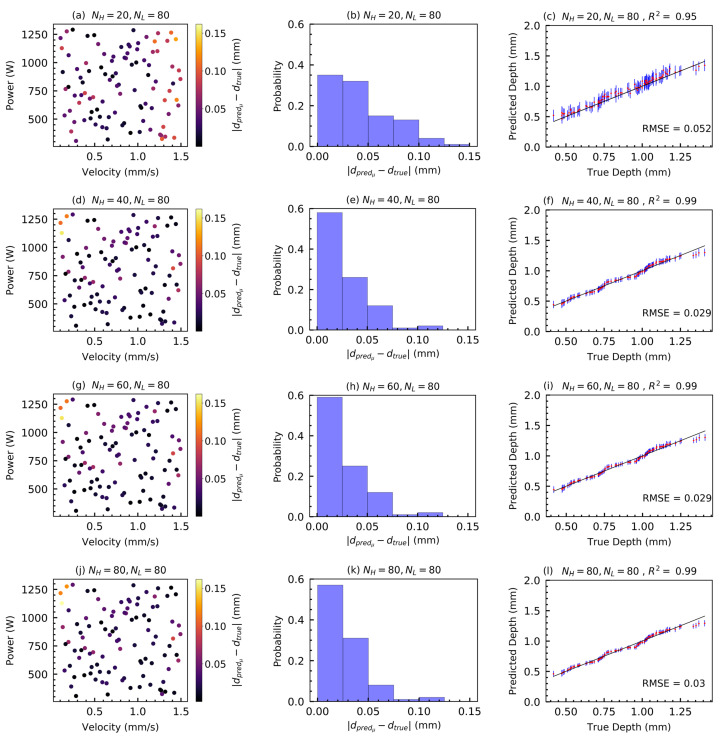
Effects of adding HF data to MFGP surrogates. (**a**,**d**,**g**,**j**) Variation of the absolute error $|d_{pred_{\mu }}-d_{true}|$ over $X_{test}$ as a function of $N_{H}$. $N_{H}$ is varied from 20 to 80, in steps of 20, while keeping $N_{L}$ constant at 80. Each circle on the figure indicates the error at a test input data point in $X_{test}$. (**b**,**e**,**h**,**k**) Histograms approximating the probability distribution of the absolute errors in the prediction over $X_{test}$. The bin probabilities indicate the fraction of points in the test set for which the absolute values of the prediction error lie within the respective bin limits. (**c**,**f**,**i**,**l**) Parity plots comparing the true depths at $X_{test}$ (sorted in an increasing order of magnitude) with the corresponding depths predicted by the respective MFGPs, along with the RMSEs of prediction. The predicted depth, being probabilistic, is represented by a filled circle indicating the mean (μ), and vertical bars indicating the associated uncertainty in prediction ($\mu \pm 1.0\sigma_{pred}$). The individual figure captions also include the coefficient of determination, $R^{2}$ in the predicted mean melt pool depths for the respective MFGP surrogates.

The effect of adding HF data to MFGP surrogates is shown in Figure 11. $N_{L}$ is kept fixed at 80 for all MFGPs. The error probability distribution indicates a significant increase in prediction accuracy as $N_{H}$ is increased from 20 to 40, with almost 60% of the predictions falling in the bin corresponding to the smallest absolute prediction error for $N_{H}=40$. This probability is calculated as $p[0\;mm\leq |d_{pred_{\mu }}-d_{true}|\leq 0.025\;mm]$. The probability shows a slight increase with $N_{H}$ = 60, but the overall mean prediction performance appears to saturate after $N_{H}$ = 40, when a coefficient of determination, $R^{2}$ = 0.99 and RMSE of 0.029 are achieved. Adding more HF data shows little effect in the mean prediction, as evinced by the respective $R^{2}$ and RMSE scores. The uncertainty in prediction, as expected, progressively decreases with the addition of HF data.

**Figure 12:**
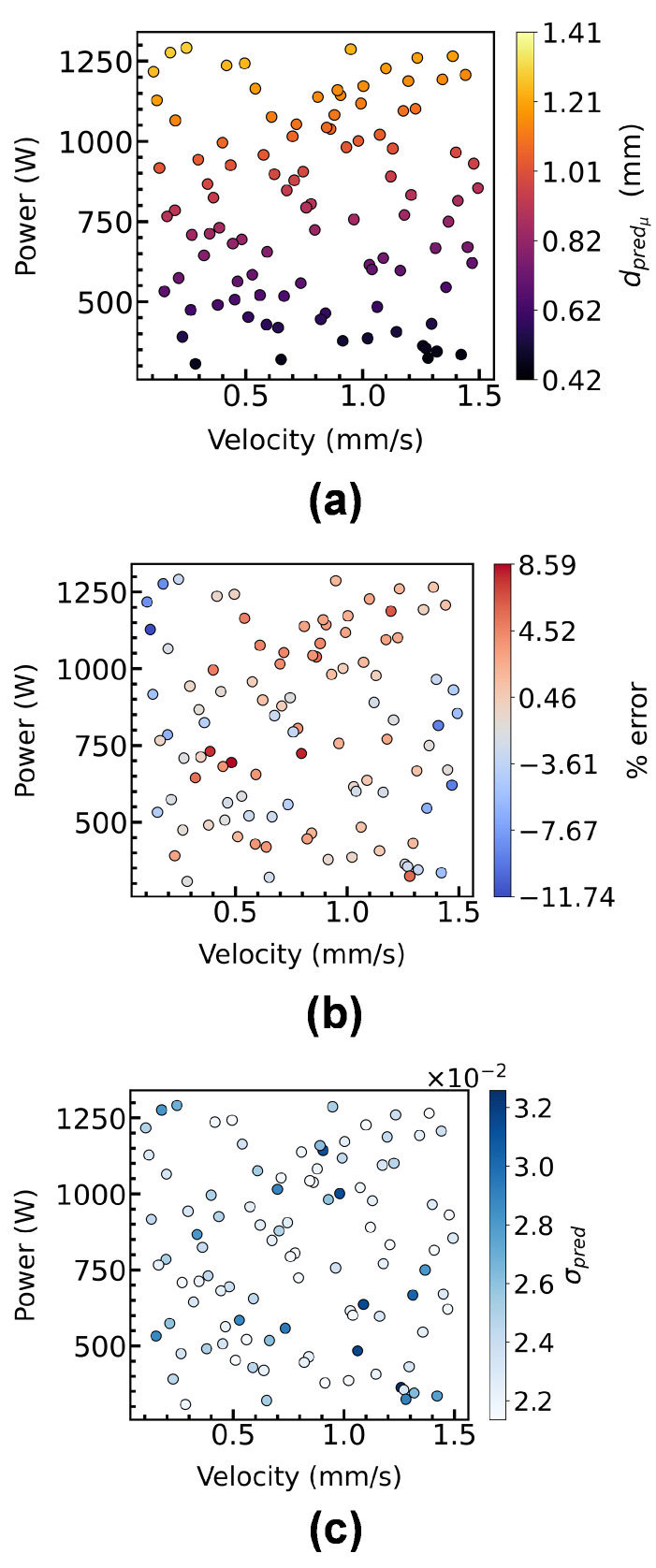
Comparison of (**a**) the mean predicted melt pool depth with (**b**) % error calculated as ($\frac{d_{pred_{\mu }}-d_{true}}{d_{true}})\times 100\%$, and (**c**) standard deviation, $\sigma_{pred}$ associated with each prediction, for MFGP with $N_{H}=40$ and $N_{L}=80$ on $X_{test}$ of 100 randomly sampled input data points from $X_{Space}$.

Figure 12 shows the predicted melt pool depths at $X_{test}$ along with the prediction error % and the standard deviation associated with it obtained via an MFGP with $N_{H}$ = 40 and $N_{L}$ = 80 that results in the high $R^{2}$ and low RMSE values reported for the same case in Figure 11f. The results show that a maximum number of 40 HF input-output data points and 80 LF input-output data points is enough to develop a robust MFGP surrogate to predict the melt pool properties (e.g., melt pool depth) for the case studied in this work.

**Figure 13:**
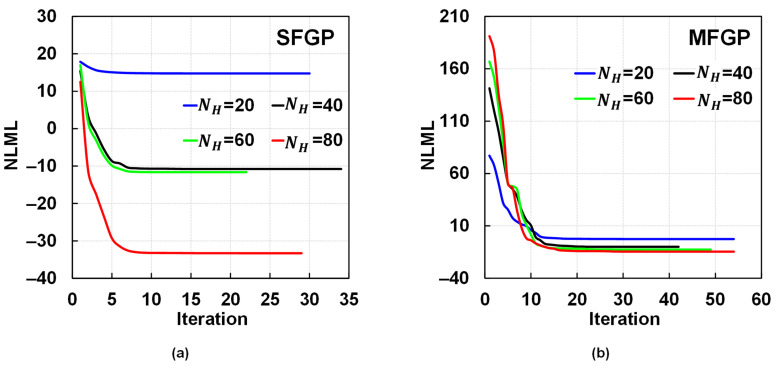
Comparison of convergence of NLML: (**a**) SFGP for 20, 40, 60, and 80 HF points and (**b**) MFGP for 20, 40, 60, and 80 HF points integrated with 80 LF points.

NLML is minimized using the L-BFGS optimization scheme. Figure 13 shows the NLML convergence curves for SFGP and MFGP for each case of $N_{H}$ where the MFGP surrogate is augmented with 80 LF points. The SFGP surrogate is found to be optimized with a lower number of iterations compared to the MFGP surrogate, for all cases of $N_{H}$. However, the MFGP surrogate converges to a better optimum for cases with a lower number of HF predictions, i.e, $N_{H}=20$, 40, and 60. A lower value of NLML corresponds to a better optimum and, hence, an improved prediction by the surrogate. The MFGP convergence curves are less susceptible to variations in the number of HF predictions, unlike SFGP where large disparities in the optimum values are observed with a change in $N_{H}$. Naturally, SFGP converges to a better optimum for $N_{H}=80$ since it is being trained with more HF predictions, that are closer to the true values, without any influence from the less accurate LF predictions. The results bolster the robustness and reliability of MFGP especially in the data-scarce regime.

### 3.3. Design of Inverse Process Maps

While the forward process maps including UQ are extremely critical in identifying the effects of process parameters on the melt pool depths, it is not sufficient for intelligent process planning. AM is a spatio-temporally evolving process because of which the thermal conditions continuously change as a part is built-in layers [37]. This can cause significant disparities in the microstructures and properties of the first and the last layers [59]. The melt pool depths vary across different layers and tracks due to the progressive addition of thermal energy to the part during the build process. Non-uniform melt pool depths in AM are widely reported in the open literature involving experimental [60,61] as well as numerical [37,62] investigations. The development of inverse process maps is, therefore, a critical requirement for achieving a consistent melt pool depth throughout the build process, even when the thermal conditions change continuously [63]. The inverse process maps are developed by solving an optimization problem that searches for process parameters to maintain the melt pool depth at the desired value during a single-layer single-track L-DED process. Since an integration of the HF model with an optimization tool will be computationally expensive, this paper proposes to develop the inverse process maps in an MF setting, whereby the MFGP surrogates are used to solve an optimization problem under a limited (pre-defined) computational budget.

The MFGP approach described in the previous subsection is extended in the setting of BO to minimize an objective function: $J\left(t,P,v\right)≜\frac{|d\left(t,P,v\right)-d^{*}|}{|d^{*}|}$, where d(t,P,v) is the melt pool depth obtained at discrete time instants during a representative build process for a given choice of process parameters (P,v), and $d^{*}$ is the desired melt pool depth. The total duration of the build process is discretized into time intervals Δt, during which the process parameters are kept constant at the optimized values of the previous time step. Making the Δt finer would potentially allow for a smoother variation of process parameters during the build process. It is easily understood that J(t,P,v) is minimized when $d\left(t,P,v\right)=d^{*}$ at all time instants under consideration, and hence, solving the optimization problem amounts to finding process parameters (P,v) that maintain the melt pool depth close to $d^{*}$.

**Figure 14:**
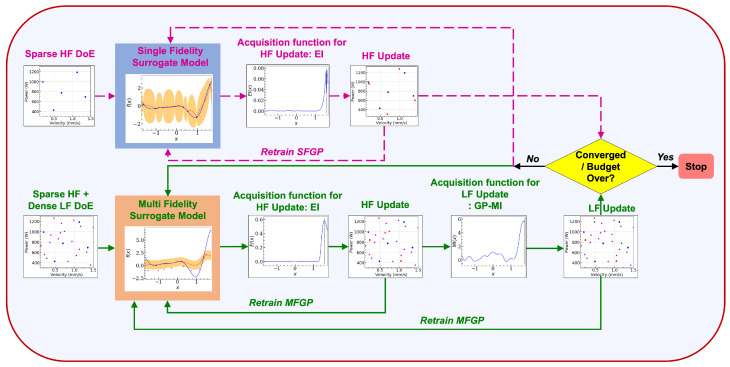
Framework of the MFGP and the SFGP Bayesian Optimization routines. The solid green line indicates the path of MFGP-BO and the dashed magenta line indicates the path of SFGP-BO.

The MFGP-BO optimization algorithm is schematically shown in Figure 14 and formulated as follows:

1. First, an initial MFGP surrogate is learned with $N_{H}$ number of HF input-output data points and $N_{L}$ number of LF input-output data points. LHS is employed to select $N_{L}$ input data points in the process parameter space, $X_{space}$, for which the LF model is used to obtain the melt pool depths. Similarly, $N_{H}$ input data points are selected in the process parameter space, $X^{*}\in X_{space}$, through a separate LHS, for which the melt pool depths are obtained from the HF model. It is to be noted that $N_{H}<<N_{L}$.
2. A prospective HF input data point is selected from the search space $X_{space}$, using the EI acquisition function [48], and the corresponding output data point is obtained from the HF model. This input-output data is added to the initial MFGP surrogate.
3. The MFGP surrogate is then retrained, and a prospective LF input data point is selected from $X^{*}$ using the GP-MI acquisition function [49], and the corresponding output data point obtained from the LF model. Thereafter, this input-output data is added to the MFGP surrogate, followed by another step of surrogate retraining.
4. This sequential selection of new HF and LF data, followed by surrogate retraining (Steps 2 and 3) is performed until the optimization budget expires. The optimization budget limitation is manifested by a maximum allowable $N_{\mathrm{opt}}$, denoting the number of sequential optimization steps that are allowed to be performed, and is a user-defined parameter.

The optimization framework involving the SFGP surrogate is similar, albeit it only involves the HF model as the sole fidelity level. Hence, the SFGP optimization algorithm starts with $N_{H}$ number of HF data for training the surrogate. LHS is employed to select $N_{H}$ input data points in the process parameter space, $X_{space}$, for which the HF model is used to obtain the melt pool depths. The selection of new HF input data in $X^{*}\in X_{space}$ is performed using the EI acquisition function in BO. The output data is obtained from the HF model. Thereafter, the input-output data is added back to the initial SFGP surrogate for retraining it, and the sequence continues till the optimization budget expires. Similar to the regression studies, the optimizations are also carried out over 50 initializations, and the results reported are the average over these initializations. Since the MFGP surrogate is used for optimization, analyzing its performance over multiple initializations ensures that the performance metrics do not reflect a bias inadvertently introduced by the choice of the initial MFGP surrogate. For example, if an initial MFGP caters to a local cluster in the search space of process parameters, the surrogate would likely be poor in other areas of the input domain. One way to counter that problem is to have an initial input-output training data set that is well spread out, e.g., using separate LHSs for selecting $N_{H}$ and $N_{L}$ input points from $X_{space}$. However, if the initial MFGP surrogate contains input points that are close to the true optimal point, there may be a tendency for the optimization routine to converge quickly to the global optima.

#### 3.3.1. Performance Metrics

The MFGP optimization framework is compared with its SFGP counterpart with respect to computational savings and the quality of the optimized process parameters. The optimization performance of both are based on the average performance over multiple initializations of the initial DoE to avoid any bias arising from the choice of training data.The optimization routine is executed only if the initial MFGP/ SFGP surrogate has no (P,v) input for which the melt pool depth is within $\left(d^{*}\pm \epsilon \right)$ for a pre-defined ϵ>0. This ensures that no optimization routine gets accidentally *fortunate* with an initial LHS input data point yielding close to the desired melt pool depth. The performance metrics are: (1) Fraction of optimization budget consumption ($\chi_{\mathrm{budget}}$) and (2) Quality Improvement (QI). The metrics are defined as follows:

1. $\chi_{\mathrm{budget}}=\frac{N^{*}/N_{T}}{N_{\mathrm{opt}}}$, where $N^{*}=\sum_{i=1}^{N_{T}}N_{d_{i}∼d^{*}}$. Here, $N_{d_{i}∼d^{*}}$ indicates the optimization iteration number at which the obtained depth $d_{i}$ is closest to $d^{*}$ in absolute norm, $N_{T}=$ number of initializations out of 50 for which the optimization routine is executed, according to the ϵ criterion described above. Hence, $N^{*}/N_{T}$ indicates the average number of optimization steps required to obtain the process parameters for which the melt pool depth is closest to the desired value. Normalizing $N^{*}/N_{T}$ with $N_{\mathrm{opt}}$, thus, reflects the fraction of the optimization budget that is consumed.
2. QI = $\frac{{RMSE}_{\mathrm{SFGP}}-{RMSE}_{\mathrm{MFGP}}}{{RMSE}_{\mathrm{SFGP}}}\times 100\%$ where, ${RMSE}_{\mathrm{MFGP}/\mathrm{SFGP}}=\sqrt{\frac{\Sigma {\left(d_{i}-d^{*}\right)}^{2}}{N_{T}}}$where $d_{i}$ is the melt pool depth (mm) closest to $d^{*}$ in absolute norm obtained within $N_{\mathrm{opt}}$ iterations for each initialization, $d^{*}$ is the desired depth (mm). The subscripted MFGP/SFGP denote the RMSE obtained from the MFGP/SFGP surrogates respectively. QI is a measure of comparing the process parameter combinations obtained from MFGP-BO and SFGP-BO with respect to the closeness of the respective melt pool depths to the desired depth.

#### 3.3.2. Optimization Performance—SFGP-BO vs. MFGP-BO

**Figure 15:**
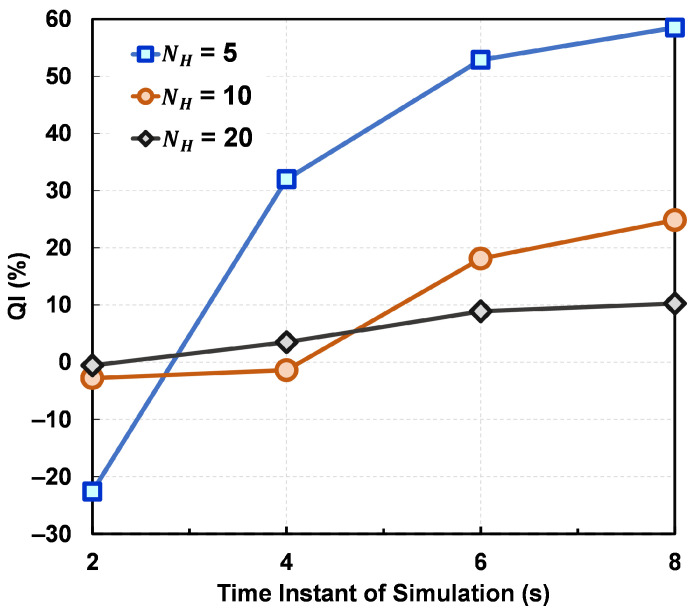
Quality improvement (QI) compared for the three $N_{H}$ values. $N_{\mathrm{opt}}$ = 5 has been chosen for all optimization exercises.

The comparison of QIs among different surrogates (Figure 15) shows that the quality of the optimized design points obtained from MFGP tends to be better than those obtained from SFGP. QI is the highest with $N_{H}=5$ and the lowest with $N_{H}=20$ for all time steps. This is expected since adding HF data points in the initial DoE allows the SFGP surrogate to learn the response surface better in the input-output space, which results in better predictions from the SFGP optimization. This analysis shows that MFGP surrogates can significantly improve the optimization performance particularly in the scarce-data regime.

**Figure 16:**
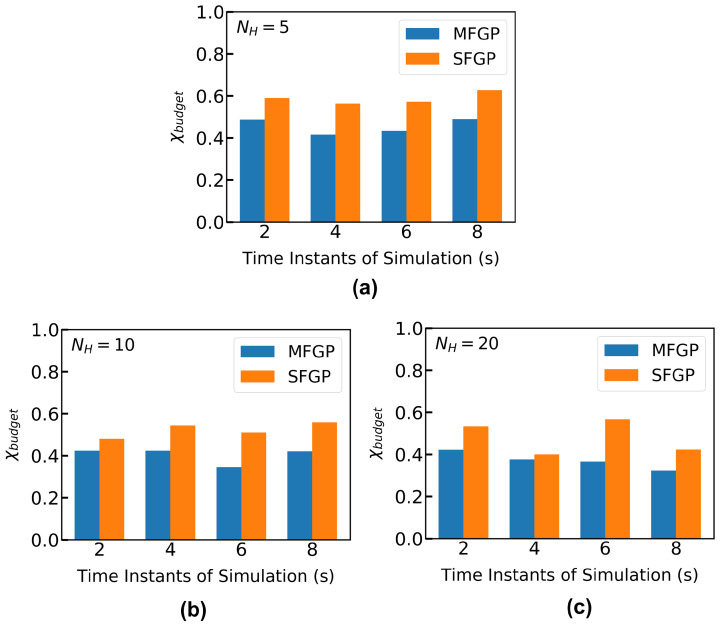
Optimization performance metrics for maintaining the melt pool depth at $d^{*}=1$ mm. $d^{*}=1$ mm is selected based on the work by Toyserkani and Khajepour [61]. The search space, $X^{*}$ consists of 1000 randomly sampled input data points from $X_{space}$. For all MFGP surrogates, $N_{L}/N_{H}=4$ is maintained. The optimization is performed starting from *t* = 2 s till *t* = 8 s at every 2 s time interval. The maximum time is 8 s since the melt pool depth reaches a steady-state value within 8 s for the selected geometry and the process parameter range. The optimization is performed when there are no initial melt pool depth values in the initial MFGP surrogate within 1±0.03 mm (ϵ=30μm). A comparison of $\chi_{\mathrm{budget}}$ for MFGP and SFGP with (**a**) $N_{H}=5$ (**b**) $N_{H}=10$ and (**c**) $N_{H}=20$. $N_{\mathrm{opt}}$ = 5 is chosen for all optimization exercises.

Figure 16 shows the comparison of the optimization performance between MFGP and SFGP. Low values of initial $N_{H}$ ($N_{H}<40$) are selected for this comparison, since it is previously observed from Figure 11 that $N_{H}=40$ points result in a highly accurate fit in the input-output space under consideration, and, hence, is expected to perform well in the optimization phase. The true potential of the optimization algorithm is, therefore, tested when the initial HF information is not significant enough to start with a good response surface of the objective function. Such a scarcity of data is a much closer representation of the real-world design optimization tasks when dealing with very expensive process models. From Figure 16a–c, it is observed that the MFGP surrogate results in lower $\chi_{\mathrm{budget}}$ for all $N_{H}$ selections at several discrete time instants. This indicates, on an average, around 12% reduction in the consumption of the optimization budget is observed for the cases investigated in this paper. This indicates the benefit of integrating LF information through MFGP surrogates to identify optimal points faster than SFGP surrogates that solely use the HF information.

## 4. Conclusions and Future Work

This paper has developed a methodology using MFGP and MFGP-BO for designing forward and inverse temporal process maps in L-DED. The continuous changes in the melt pool geometry are predicted by a low-cost MFGP surrogate developed by integrating two melt pool simulation models. The LF model is based on the analytical Eagar-Tsai’s model while the HF model is based on Autodesk Netfabb^®^’s FEM model. The uncertainties associated with the predictions of the melt pool depths are quantified using GPs. It is demonstrated that MFGP predictions are more accurate and have higher confidence than SFGP. Once the temporal forward process maps are developed, MFGP and SFGP are coupled with BO for developing the inverse process maps. These maps are used to estimate the process parameters required to achieve the desired melt pool depth. The BO algorithm minimizes an appropriate objective function that quantifies the deviation of the melt pool depth from the desired value under computational budget constraints to yield the optimal process parameters under varying thermal conditions.

The reliability of the optimization algorithm, however, depends on the fundamental physics addressed by the models. While the HF models are more capable in resolving the fundamental physics, the computational cost involved in the optimization process can be significantly high. For example, the cost of running a Netfabb^®^ L-DED model for a single-track and single-layer simulation as described in this paper is ∼10 times higher than the Eagar-Tsai’s model at *t* = 2 s, and ∼150 times higher at *t* = 10 s. The MFGP-BO, where the HF model is integrated with the LF model, thus fairs better than the SFGP-BO by reducing the computational overhead by 12% percent without compromising on the quality of the optimized process parameters. Such a benefit will continue to increase for larger domain sizes having multi-layer multi-track depositions. Hence, this algorithm is particularly conducive for process planning purposes in data-scarce regimes.

The demonstrations of MFGP and MFGP-BO are presented, in this paper, for designing the forward and inverse temporal process maps, respectively, incorporating UQ for the melt pool depth. However, the formulation is flexible to accommodate for other properties such as the residual stress or the mean grain size as long as multiple process models having different fidelities exist. The formulation can also incorporate more than two levels of fidelity by appropriately modifying the covariance matrix which determines the correlations among the different levels of fidelity [15]. The realization of such a formulation has the potential to reduce the requirement for extensive computational investigation toward the development of sophisticated model-based feedforward and feedback control strategies [64] in and beyond L-DED AM. Additional investigations are also planned in the future as summarized below:

1. Developing multi-dimensional process maps that include other process parameters such as scan spacing, powder feed rate, and build plate temperature.
2. Augmenting the present two-fidelity surrogate by incorporating experimental data that would serve as the highest-fidelity level (true values).
3. Using the MF surrogate for constrained optimization e.g., estimating the optimal parameters for controlling the melt pool depth while being constrained to maintain the desired microstructure (e.g., % of equiaxed or columnar grain morphology).
4. Formulating an MF framework that allows for the inclusion of heterogeneous input spaces across different fidelities. For example, the HF model can take multiple process parameters e.g., scan pattern, hatch spacing, etc. while the LF model can incorporate only the primary process parameters, *P* and *v*. Optimization with such different input parameter space needs special methods such as heterogeneous transfer learning [65] to learn from a common subspace of the inputs.

## Figures and Tables

**Table 1:** Melt pool dimensions calculated using Eagar-Tsai for *P* = 1000 W and *v* = 1 mm/s.

Time (s)	Melt Pool Depth (mm)	Melt Pool Width (mm)	Melt Pool Length (mm)
2	1.238	2.751	2.792
4	1.334	2.934	2.994
6	1.375	3.012	3.083
8	1.397	3.056	3.133
10	1.410	3.083	3.164

**Table 2:** Temperature dependent properties for CMSX-4^®^.

T ${}^{\circ }$C	Density (g/cm^3^)	Thermal Conductivity (W/(m.K))	Specific Heat (J/(g.K))
27	8.592	11.902	0.426
127	8.552	12.655	0.454
327	8.49	14.794	0.53
527	8.41	16.875	0.542
727	8.318	19.274	0.589
927	8.211	22.226	0.652
1127	8.079	26.922	0.795
1327	7.921	35.331	0.62
1527	7.396	35.395	0.693
1727	7.208	38.915	0.698

## Data Availability

The datasets generated during and/or analyzed during the current study are available from the corresponding author on reasonable request.

## Abbreviations

The following abbreviations are used in this manuscript:

AL	Active Learning
AM	Additive Manufacturing
BO	Bayesian Optimization
DoE	Design of Experiments
EI	Expected Improvement
FEM	Finite Element Modeling
GP	Gaussian Process
HF	High Fidelity
L-BFGS	Limited Memory Broyden-Fletcher-Goldfarb-Shanno
L-DED	Laser-Directed Energy Deposition
LF	Low Fidelity
LHS	Latin Hypercube Sampling
MF	Multi Fidelity
MFGP	Multi Fidelity Gaussian Process
MFGP-BO	Multi Fidelity Gaussian Process—Bayesian Optimization
MI	Mutual Information
NLML	Negative Log Marginal Likelihood
SF	Single Fidelity
SFGP	Single Fidelity Gaussian Process
SFGP-BO	Single Fidelity Gaussian Process—Bayesian Optimization
UQ	Uncertainty Quantification
Nomenclature	
$\alpha_{L}$	Absorptivity of laser beam
δ(x)	Discrepancy function of x
ρ	Correlation function
$\theta_{1},\theta_{2}$	Hyperparameters of the covariance
*q*	Heat flux
μ(x)	Mean function of x
$\chi_{\mathrm{budget}}$	Fraction of the optimization budget that is consumed
cholesky(A)	Cholesky decomposition: *L* is a lower triangular matrix
	such that $LL^{T}=A$
$\rho_{p}$	Material density
ϵ	Pre-defined tolerance in optimized value
σ(x)	Variance function of x
$\sigma_{L}$	Distribution parameter
$\sigma_{pred}$	Standard deviation of the posterior predictive Gaussian distribution
	of the melt pool depth
${\sigma_{\epsilon }}^{2}$, ${\sigma_{\epsilon_{1}}}^{2}$, ${\sigma_{\epsilon_{2}}}^{2}$	Noise variance
$a_{p}$	Thermal diffusivity of material
$c_{p}$	Specific heat of material
*d*	Melt pool depth
$d_{i}$	Melt pool depth closest to desired depth at *i*th initialization
	for which optimization routine is executed
$d^{*}$	Desired melt pool depth
$d_{L},d_{H}$	Predicted melt pool depth from LF and HF models, respectively
$d_{true}$	True melt pool depth
$d{}_{pred}{}_{\mu }$	Mean of the posterior predictive
	Gaussian distribution of the melt pool depth
E(y)	Expectation of y
f(x)	Gaussian process function values, $f=(f\left(x_{1}\right),\cdots ,f\left(x_{n}\right))$
$f_{x^{*}}$	Gaussian process (posterior) prediction (random variable)
${\hat{f}}_{x^{*}}$	Gaussian process posterior mean
$h_{\mathrm{eff}}$	Effective heat transfer coefficient
$h_{\mathrm{forced}}$	Forced convection heat transfer coefficient
$h_{\mathrm{free}}$	Free convection heat transfer coefficient
$h_{\mathrm{radiation}}$	Radiation convection heat transfer coefficient
*J*	Objective function for BO
$k,k(x,x^{\prime })$	Kernel functions of GPs
$k_{2},k_{1}$	Kernel functions of HF and LF GPs
K	Covariance matrix
$k_{p}$	Thermal conductivity of material
$N_{L},N_{H}$	Number of LF points, Number of HF points
$N_{\mathrm{opt}}$	Maximum allowable optimization iterations
$N_{T}$	Number of initializations for which the optimization
	routine is executed
$N^{*}$	Sum of optimization iteration numbers at which the
	obtained depth is closest to $d^{*}$ in absolute norm
$N_{d_{i}∼d^{*}}$	Optimization iteration number at which
	the obtained depth $d_{i}$ is closest to $d^{*}$ in absolute norm
*P*	Laser power
$P^{trn},v^{trn}$	Training input data: Laser power, velocity
$P^{tst},v^{tst}$	Test input data: Laser power, velocity
QI	Quality Improvement
RMSE	Root mean square error
$RMSE_{avg}$	RMSE averaged over all initializations
$RMSE_{MFGP},RMSE_{MFGP}$	RMSE calculated for MFGP-BO,RMSE calculated for MFGP-BO
$R^{2}$	Coefficient of Determination
*t*	Time
$t^{\prime }$	Dummy integration variable
$t_{LF},t_{HF}$	Time taken by LF model (s), Time taken by HF model (s)
$T_{0}$	Initial temperature
Δt	Time step
$T_{s}$	Surface temperature
$T(x_{c},y_{c},z_{c},t$)	Temperature as a function of coordinates ($x_{c},y_{c},z_{c}$) and time (*t*)
$T_{\infty }$	Ambient temperature
$T_{L}$	Liquidus temperature
*v*	Laser scan velocity
$\Sigma^{tst}$	Covariance of the posterior Gaussian distribution at test input
$\mu^{tst}$	Mean of the posterior Gaussian distribution at test input
$x,x^{\prime }$	Input variables
$\hat{x}$	Value of x that maximizes an objective function
**x**${}^{trn}$, **x**${}^{tst}$	Training input, Test input
$x_{2},x_{1}$	Inputs to HF and LF models
X	Combined input to a GP consisting of $x_{2},x_{1}$
$X_{Space}$	Process parameter space
$X_{Test}$	Test space
$X^{*}$	Search space for optimization
$y,y^{\prime }$	Output of a GP
Y	Combined output of a GP consisting of $y_{2},y_{1}$
$y_{2},y_{1}$	Outputs from HF and LF models
**y**${}^{trn}$, **y**${}^{tst}$	Training output, Test output
V	Variance
N	Gaussian distribution
$\alpha ,\Psi ,\psi_{1}\;\psi_{2},\beta$	Intermediate parameters in Algorithm 1
